# The survival outcomes and prognostic factors of hepatocellular carcinoma among type 2 diabetes patients: a two-centre retrospective cohort study

**DOI:** 10.55730/1300-0144.5498

**Published:** 2021-12-02

**Authors:** Noor Atika AZIT, Shahnorbanun SAHRAN, Leow VOON MENG, Manisekar K. SUBRAMANIAM, Suryati MOKHTAR, Azmawati MOHAMMED NAWI

**Affiliations:** 1 Department of Community Health, Faculty of Medicine, National University of Malaysia, Kuala Lumpur, Malaysia; 2 Kuala Muda District Health Office, Ministry of Health Malaysia, Kedah, Malaysia; 3 Centre for Artificial Intelligence Technology (CAIT), Faculty of Information Science and Technology, National University of Malaysia, Selangor, Malaysia; 4 Department of Clinical Medicine, Advanced Medical and Dental Institute (AMDI), University of Science, Malaysia, Penang, Malaysia; 5 Department of Surgery, Hospital Sultanah Bahiyah, Ministry of Health Malaysia, Kedah, Malaysia; 6 Department of Surgery, Hospital Selayang, Ministry of Health Malaysia, Selangor, Malaysia

**Keywords:** Hepatocellular carcinoma, type 2 diabetes mellitus, liver neoplasm, prognosis, retrospective studies

## Abstract

**Background/aim:**

To determine the survival outcomes and prognostic factors associated with hepatocellular carcinoma (HCC) survival in type 2 diabetes (T2D) patients.

**Materials and methods:**

This was a retrospective cohort study involving two hepatobiliary centres from January 1, 2012, to June 30, 2018. Medical records were analysed for sociodemographic, clinical characteristics, laboratory testing, and HCC treatment information. Survival outcomes were examined using the Kaplan–Meier and log-rank test. Prognostic factors were determined using multivariate Cox regression.

**Results:**

A total of 212 patients were included in the study. The median survival time was 22 months. The 1-, 3-, and 5-year survival rates were 64.2%, 34.2%, and 18.0%, respectively. Palliative treatment (adjusted hazard ratio [AHR] = 2.82, 95% confidence interval [CI] 1.75–4.52), tumour size ≥ 5 cm (AHR = 2.02, 95%CI: 1.45–2.82), traditional medication (AHR = 1.94, 95%CI: 1.27–2.98), raised alkaline phosphatase (AHR = 1.74, 95%CI: 1.25–2.42), and metformin (AHR = 1.44, 95%CI: 1.03–2.00) were significantly associated with poor prognosis for HCC survival. Antiviral hepatitis treatment (AHR = 0.54, 95% CI: 0.34–0.87), nonalcoholic fatty liver disease (NAFLD) (AHR = 0.50, 95% CI: 0.30–0.84), and family history of malignancies (AHR = 0.50, 95%CI: 0.26–0.96) were identified as good prognostic factors for HCC survival.

**Conclusion:**

Traditional medication, metformin treatment, advanced stage and raised alkaline phosphatase were the poor prognostic factors, while antiviral hepatitis treatment, NAFLD, and family history of malignancies were the good prognostic factors for our HCC cases comorbid with T2D.

## 1. Introduction

Hepatocellular carcinoma (HCC) is a primary malignancy of the liver and is one of the major leading causes of cancer death globally. The 2020 global cancer statistics revealed increased mortality in liver cancer (LC) from 2019 [1]. LC now ranks third among all-tumour type mortality after lung and colorectal cancer [1,2]. Type 2 diabetes (T2D) had been identified as an independent risk factor for HCC, with a double relative risk of HCC mortality [3,4]. The pathogenesis of HCC in T2D may involve cirrhotic and noncirrhotic pathways [3]. Among the important factors contributing to tumour proliferation and survival in T2D are hyperglycaemia, insulin resistance, hyperinsulinemia, intestinal dysbiosis, chronic inflammation, and increased oxidative stress [5–7]. These multicarcinogenesis pathways had contributed to elevated risk of HCC in T2D patients from the nondiabetes mellitus population. Moreover, the survival outcomes of HCC were also influenced by factors such as the tumour burden, clinical characteristics at diagnosis, treatment, and the sociodemographic determinants [8–11].

In previous epidemiological studies, HCC was found to have a lower survival rate in diabetes mellitus (DM) patients [4,12]. For example, in a large cohort study conducted in Taiwan, the survival rate of T2D patients with HCC was found significantly lower than the non-DM patients. The 1-, 3-, and 5-year survival rates were 56.8%, 26.4%, and 12.7% in DM patients, respectively, compared to non-DM patients at 61.6%, 32.8%, and 18.8%, respectively [10]. The survival rate of HCC was also highly connected with the country’s development. Diseases like HCC necessitate more advanced health care, with higher-quality care being more easily available in more developed states. Moreover, socioeconomic status significantly influences a country’s healthcare capabilities and health-seeking behaviour among the population [11].

Therefore, the rising of HCC related to T2D is a public health concern. The trend has been observed in many low-HCC regions, e.g., Europe, Northern America, Australia, New Zealand, and South America [1,13]. As the prevalence of these risk factors increases, HCC incidence and mortality rates are projected to grow further, increasing the healthcare system’s burden, especially among the developing countries.

Malaysia is a developing country with a high DM prevalence [14,15]. The prevalence is increasing in Malaysia and is expected to increase to 31% by 2025 [16]. This situation has created a challenge for disease control and prevention programs as the population grows and ages. Currently, LC is the second highest cause of cancer death in Malaysia, with most (74.3%) of cases presenting at stage 4 disease [17]. Understanding the survival outcomes and factors associated with HCC prognosis will benefit future T2D and HCC management strategies. However, most studies on HCC survival outcomes among T2D patients have mainly been conducted in developed countries, e.g., New Zealand, Taiwan, and Japan [4,18,19]. Therefore, we conducted this study to understand the survival outcomes and associated prognostic factors for HCC survival among T2D patients to benefit the disease control and prevention programs.

## 2. Materials and methods

### 2.1. Study site and ethics statement

This study was conducted at Hospital Selayang (HS) and Hospital Sultanah Bahiyah (HSB), which are among the five designated hepatobiliary referral centres in Malaysia. HS is the national referral centre of the hepatobiliary subspecialty. Study approval was obtained from the Malaysian Ministry of Health Medical Research and Ethics Committee (NMRR-18-3704-45037) and the National University of Malaysia Faculty of Medicine Ethics Committee (JEP-2019-356), including the exemption of the requirement for informed consent.

### 2.2. Study design and study population

This was a retrospective cohort study for determining the survival outcomes and prognostic factors associated with HCC survival among T2D patients.

**Figure 1:**
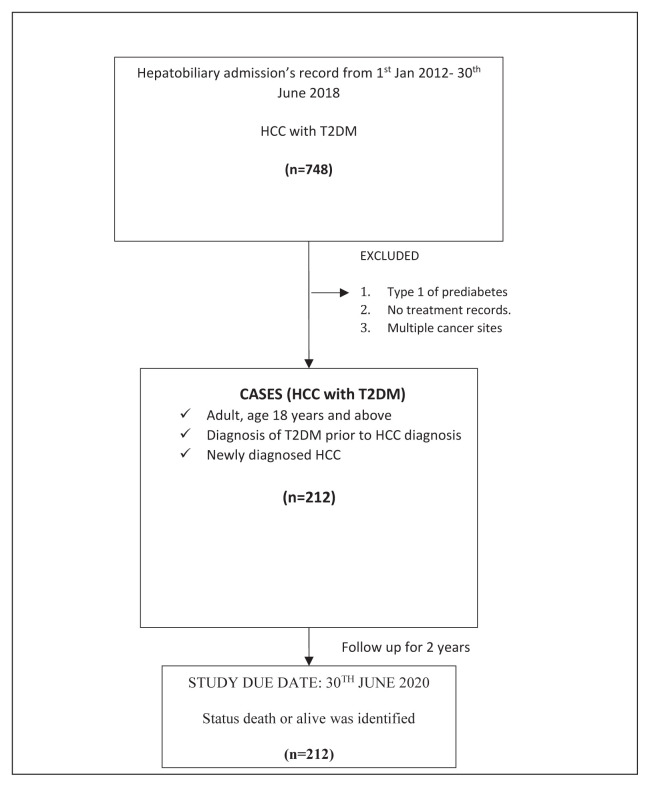
The overall study flow. A total of 748 patients with HCC with T2D were extracted from the medical record database from January 1, 2012, until June 30, 2018. Two hundred and twelve patients met the inclusion and exclusion criteria. All subjects were followed up from July 1, 2018, until June 30, 2020, for the survival status.

A total of 212 adults (aged ≥ 18 years), newly diagnosed with HCC and with a prior diagnosis of T2D were selected via convenience sampling from the HS and HSB hepatobiliary departments from January 1, 2012, to June 30, 2018. Type 1 diabetes, prediabetes, patients without diabetic treatment records and multiple cancer sites were excluded. All participants were followed until June 30, 2020 (2 years), and their status, i.e. dead or alive, was determined from medical records and verified with National Death Registry data. The sociodemographic data, clinical characteristics, medical investigations (biochemical parameters and imaging), and treatment data were extracted from the hospitals’ electronic medical records. The sample size was calculated using Power and Sample Size Calculations software, version 3.1; the power of 80% at a 95% confidence interval (CI) with reference to a previous study [20] of a minimum of 63 events was met. Figure 1 shows the overall study design flow.

### 2.3. Study variables

We reviewed the electronic medical records from both hospitals to obtain the studied variables. Death statuses were verified with the National Death Registry.

### 2.4. Outcome variables

The survival time was defined as the time from the date of HCC diagnosis until death (months), loss to follow-up, or censoring (months). The survival outcome was the status (dead, alive) at the study due date.

### 2.5. Independent variables

Sociodemographic and clinical characteristics were categorized into “yes” or “no”. The sociodemographic characteristics were age, sex, race. The clinical characteristics were: weight loss, lethargy, loss of appetite, abdominal pain or discomfort, jaundice, viral hepatitis, nonalcoholic fatty liver disease (NAFLD), cirrhosis, portal hypertension, hypertension, overweight/obesity, history of blood transfusion, family history of malignancies, metformin, sulfonylureas, insulin, statins, antivirals for hepatitis, traditional medication (recorded history of taking any nonprescribed traditional medicine in the clinical notes), history of alcohol consumption, smoking, duration of diabetes (≥10 years, <10 years), and glycated haemoglobin (HbA1c) level (≥8.5%, <8.5%).

The biochemical profiles measured were full blood count, liver function, coagulation profile (international normalized ratio, INR; >1.2, ≤1.2), and alpha-fetoprotein (AFP) level (ng/mL; <20, ≥20).

HCC characteristics and treatment were Child-Pugh score (CPS) [21] at HCC diagnosis, maximum tumour diameter (cm, 0–4, ≥5), and HCC treatment (transarterial chemoembolization [TACE], surgical resection [SR]/radiofrequency thermal ablation [RFA], palliative).

### 2.6. Data analysis

All statistical analyses were performed using IBM SPSS v. 21 (IBM Corp., Armonk, NY, USA).

### 2.7. Missing data

As many retrospective studies commonly describe missing data, we applied careful missing data processing. Variables with >20% missing values were not included in the analysis. Multiple imputations were used for the included variables to preserve the study’s statistical power as this method has been proven to avoid bias compared to complete case analysis [22]. Little’s MCAR test was used to determine the randomness of the missing data [23]. Five imputed datasets were generated using a fully conditional specification algorithm for the inferential analysis. The missing data analysis is presented as the supplement materials.

### 2.8. Descriptive analysis

The descriptive characteristics of the dead and alive groups are presented as frequencies and percentages.

### 2.9. Univariate and multivariate analysis

Median survival time and 1-, 3-, and 5-year survival rate data were analysed using the Kaplan–Meier test and the log-rank test was used to compare the survival outcomes. The prognostic factors were analysed using simple and multiple Cox proportional hazards regression. Variables with p-value < 0.05 in the simple cox regression and DM-related variables (metformin, insulin, sulphonylureas, DM duration, and HbA1c) were included in the multiple Cox regression analysis. The variables were included based on their clinical importance in DM management. Multicollinearity and interactions between the variables were checked in the final model. Proportional hazard assumption was also examined.

## 3. Results

### 3.1. Descriptive and univariate analysis

A total of 212 samples were included in the analysis. The median follow-up was 23 months (interquartile range, IQR: 7, 41). At the end of follow-up, 159 patients (75%) had died, and 53 (25%) were alive. Eleven variables had missing data (0.9%–19.8%) and were missing at random (Little’s MCAR test p-value = 0.928). Table presents the frequency and percentage of the participants’ sociodemographic, clinical, and biochemical characteristics and the univariate analysis for the included variables.

### 3.2. Overall survival and factors associated with survival outcomes

The 1-, 3-, and 5-year survival rate was 64.2%, 34.2%, and 18.0%, respectively. The median survival time was 22.0 months. The variables loss of appetite, abdominal pain or discomfort, NAFLD, ascites, family history of malignancies, antiviral treatment, traditional medication, duration of T2D, CPS, maximum tumour size, HCC treatment, total bilirubin (TBil), alkaline phosphatase (ALP) level, and AFP level were significantly different (p < 0.05) between alive and dead patients.

### 3.3. Multivariate analysis

**Figure 2:**
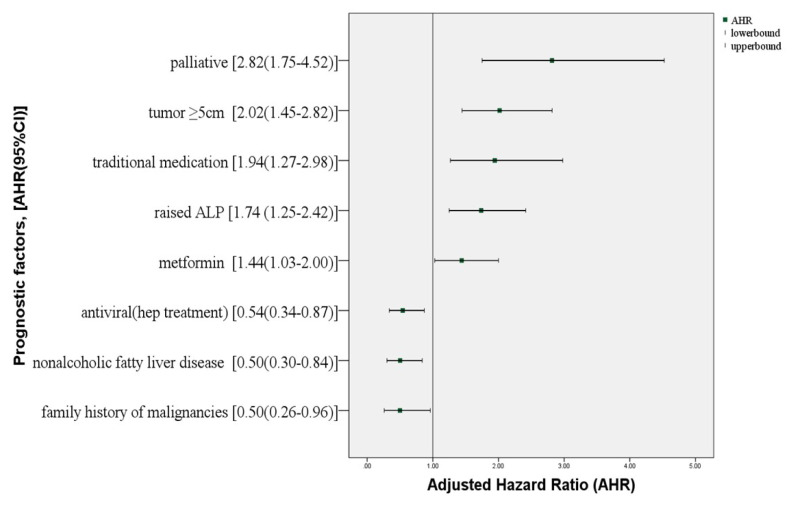
Forest plot of the prognostic factors in T2D patients with HCC.

Figure 2 shows all significant prognostic factors in the multivariate analysis. Patients with palliative treatment had a 2.82 times higher risk of death (AHR = 2.82, 95% CI: 1.75–4.52) than patients with surgical/RFA treatment. Patients with tumour size > 5 cm had 2.02 times higher risk of dying (95% CI: 1.45–2.82) than those with smaller tumours at presentation. Patients with a history of traditional medication consumption had 1.94 increased risk of death (95% CI: 1.27–2.98), and patients with raised ALP (> 129 IU/L) had 1.74 higher risk of death (95% CI: 1.25–2.42) than patients with normal ALP levels. Patients on metformin had 1.44 higher risk of death (95% CI: 1.03–2.00) than those who were not. Antiviral hepatitis treatment reduced mortality risk by 46% (AHR = 0.54, 95% CI: 0.34–0.87). In comparison to patients without NAFLD, patients with NAFLD had 50% lower risk of death (AHR = 0.50, 95% CI: 0.30–0.84), as did patients with a family history of cancer (AHR = 0.50, 95% CI: 0.26–0.96) compared to those without. There was no evidence of interaction or multicollinearity among the significant variables (variance inflation factor < 10). The proportional hazard assumption was checked using log cumulative hazard plots for all covariates.

## 4. Discussion

The present study was aimed at determining the survival outcomes and prognostic factors of HCC survival among T2D patients. Here, the 5-year survival rate was lower compared to that of developed Asian countries, e.g., Japan (30.1%), Korea (27.2%), and Singapore (24.7%) [11]. However, the rate is higher than neighbouring developing countries such as Thailand (6.9%) and China (14.1%) [11]. Many factors contributed to the differences in survival outcomes, including research methodology heterogeneity and other determinants of health. Nevertheless, the survival outcomes may indicate the cancer management system’s quality in the specific population. Our study reports a higher survival outcome than other local studies [17,24,25] because it was conducted at national hepatobiliary centres, where resources are prioritized. Therefore, we postulate that the national survival outcome is lower and requires further evaluation.

The prognostic factors antiviral therapy, NAFLD, and family history of malignancies had good predictive outcomes for HCC survival (Figure 2). In T2D patients, viral hepatitis has been found to have a synergistic effect on HCC development [26]. Nevertheless, antiviral therapy will help suppress viral replication, reducing microvascular invasion and early tumour recurrence after hepatectomy [27]. Therefore, patients with chronic hepatitis who meet the eligibility criteria should be given the option to receive treatment.

Besides, we found that NAFLD patients had a good prognostic outcome. According to the current DM management protocol, NAFLD patients undergo regular surveillance for liver disease [28]. As HCC was detected early, curative treatment for HCC can be implemented, resulting in better survival outcomes. Furthermore, a US study found that NAFLD patients with less severe liver dysfunction had more excellent overall survival rates than those with other causes of HCC following curative surgery [29]. The good survival outcomes for patients with a family history of cancer could be related to their relatives’ experience. It may help raise awareness of early HCC screening and good lifestyle behaviour to improve health outcomes.

In contrast, characteristics linked to late presentation, e.g., palliative care and larger tumour size, have been identified as poor prognostic indicators. Patients diagnosed late in their illness and are not candidates for curative treatment will be offered palliative care. A larger tumour size (≥5 cm) is one predictor of late presentation and is associated with a poor HCC survival prognosis. Many HCC staging systems use tumour size to aid treatment decision-making and cancer prognostication [30]. Patients with small tumour size, single-nodule disease, and preserved liver function are suitable for curative treatment with a 5-year survival rate of >50% [31].

Another significant discovery was that patients who used unprescribed traditional medications had a poor prognosis. Traditional medication is becoming increasingly popular among people with DM, especially in developing countries. Previous studies had reported the prevalence between 38.2% and 73.7% of traditional medication usage among DM patients [32–35]. There are two underlying reasons to affect the survival in this group. Firstly, the consumption may delay the medical treatment for HCC due to late presentation. A metasynthesis on the barrier for health-seeking behaviour among breast cancer women revealed that belief in traditional alternative care had hindered the timing of health-seeking [36]. Secondly, the hepatotoxicity of the substances or contaminants may promote HCC development, especially among the unregistered traditional products with unknown safety profiles [37,38]. Therefore, strict regulation on traditional medication marketing and usage is critical for avoiding patient harm.

Besides, only metformin was significantly associated with poor prognosis in routine DM management. Although many previous studies have revealed that metformin is a protective factor [39], other longitudinal studies have reported that it confers no survival benefit [40]. Previous research on the metformin impact found that patients using metformin had high survival outcomes in the early stages of HCC but had poor survival outcomes later in the disease [41]. Casadei Gardini et al. observed that patients with HCC on metformin and treated with sorafenib had worse survival outcomes, suggesting that the tumour may have an innate mechanism of metformin resistance due to prolonged use, which also leads to sorafenib resistance [20,42]. Furthermore, the genetic heterogeneity in the metformin pharmacokinetic response in various ethnicities is unclear [43]. Moreover, we did not explore combination therapy of metformin with multiple insulin dosages or with sulfonylureas. Insulin and metformin have antagonistic effects on HCC cells [44], explaining why the results differ from earlier research. Therefore, our findings require further evaluation.

Regarding the biochemical profiles, only raised ALP was independently associated with poor HCC survival. A metaanalysis of 21 studies on overall survival and six studies on disease-free survival found that elevated pretreatment ALP was linked to poor prognosis regardless of treatment or patient age [45]. This finding highlights the potential of ALP as a predictive biomarker in HCC prognosis.

The strength of the present study is that we determined the HCC survival outcome and prognosis factors in a perspective T2D population, where such studies are still scarce in developing countries. We discovered the essential parameters for improving cancer prevention and control programs in the future. Secondly, electronic medical record databases allowed the integration of information from all involved departments, increasing the precision of the information obtained and reducing information bias. However, some limitation of our study is the nonprobability sampling: the results may not be representative of the whole country. Nonetheless, including two hepatobiliary centres broadens the range of participants and improves the findings’ generalizability. We did not include cancer staging and the histopathological grades in the analysis because this information was unavailable in the medical records. However, the information on tumour size at presentation suggested that 45% of the patients presented at the later stage (size ≥ 5 cm) because tumour size was significantly associated with poor prognosis in HCC [30,31]. Besides, autoimmune hepatitis was also not studied due to the unavailability of the information in the medical records. Future study recommendations include studying the disease recurrence and quality of life in T2D patients with HCC.

In conclusion, understanding the prognostic factors of T2D-related HCC is essential for HCC control and prevention programs to improve the disease burden due to premature death.

## Figures and Tables

**Table:** The participants’ sociodemographic, clinical, biochemical characteristics and univariate analysis of the included variables.

Variable (N = 212)	Dead (n = 159)		Alive (n = 53)		Crude HR ^‡^	95% CI	p-value
	n	(%)	n	(%)			
Sociodemographic characteristic							
Age	67.38	(8.20) ^†^	65.47	(8.18) ^†^	1.02	1.00–1.04	0.069
Sex							0.637
Male	124	78.0	41	77.4	1.10	0.75–1.60	
Female	35	22.0	12	22.6	1.00		
Race							0.163
Chinese	76	47.8	31	58.5	1.00		
Malay	67	42.1	19	35.8	1.26	0.90–1.75	
Indian	16	10.1	3	5.7	1.59	0.92–2.72	
Clinical characteristics							
Weight loss							0.219
No	121	76.1	43	81.1	1.00		
Yes	38	23.9	10	18.9	1.26	0.87–1.81	
Loss of appetite							0.008^*^
No	120	75.5	43	81.1	1.00		
Yes	39	24.5	10	18.9	1.64	1.14–2.36	
Abdominal pain/discomfort							<0.001^*^
No	99	62.3	41	77.4	1.00		
Yes	60	37.7	12	22.6	1.87	1.35–2.59	
NAFLD							0.015^*^
No	142	89.3	40	75.5	1.00		
Yes	17	10.7	13	24.5	0.53	0.23–0.8	
Cirrhosis							0.746
No	43	27.0	17	32.1	1.00		
Yes	116	73.0	36	67.9	0.94	0.67–1.34	
Portal hypertension							0.058
No	116	73.0	47	88.7	1.00		
Yes	43	27.0	6	11.3	1.40	0.99–2.00	
Ascites							<0.001^*^
No	116	73.0	49	92.5	1.00		
Yes	43	27.0	4	7.5	2.17	1.52–3.09	
Viral hepatitis							0.073
No	83	52.2	25	47.2	1.00		
Yes	76	47.8	28	52.8	0.75	0.55–1.23	
Hypertension							0.345
No	42	26.4	12	22.6	1.00		
Yes	117	73.6	41	77.4	0.84	0.59–1.20	
Overweight/obese							0.185
No	24	24.7	13	28.9	1.00		
Yes	73	75.3	32	71.1	1.34	0.87–2.07	
History of blood transfusion							0.723
No	129	81.1	45	84.9	1.00		
Yes	30	18.9	8	15.1	1.07	0.72–1.60	
Family history of malignancies							0.005^*^
No	149	93.7	40	75.5	1.00		
Yes	10	6.3	13	24.5	0.40	0.21–0.75	
Metformin							0.201
No	39	37.1	23	43.4	1.00		
Yes	100	62.9	30	56.6	1.23	0.89–1.70	
Sulphonylureas							0.289
No	93	58.5	29	54.7	1.00		
Yes	66	41.5	24	45.3	0.84	0.62–1.16	
Insulin							0.445
No	104	65.4	37	69.8	1.00		
Yes	55	34.6	16	30.2	1.14	0.82–1.58	
Antiviral (hepatitis treatment)							0.002^*^
No	136	85.5	33	62.3	1.00		
Yes	23	14.5	20	37.7	0.49	0.32–0.77	
Statins							0.405
No	108	67.9	36	67.9	1.00		
Yes	51	32.1	17	32.1	1.15	0.83–1.61	
Traditional medicine							0.007^*^
No	131	82.4	49	92.5	1.00		
Yes	28	17.6	4	7.5	1.76	1.17–2.66	
Alcohol							0.912
No	108	67.9	37	69.8	1.00		
Yes	51	32.1	16	30.2	0.98	0.70–1.37	
Smoking							0.553
No	81	50.9	27	50.9	1.00		
Yes	78	49.1	26	49.1	1.10	0.81–1.50	
Duration of diabetes, years (n = 170)							0.060
≥10	63	51.2	20	42.6	1.41	0.99–2.01	
<10	60	48.8	27	57.4	1.00		
CPS (n = 195)							<0.001^*^
A	91	61.9	44	91.7	1.00		
B/C	56	38.1	4	8.3	1.96	1.41–2.71	
Maximum tumour size, cm (n = 190)							<0.001^*^
0–4	63	44.7	31	63.3	1.00		
≥5	78	55.3	18	36.7	1.85	1.33–2.58	
Treatment							<0.001^*^
TACE	72	45.3	28	52.8	2.86	1.82–4.51	
SR/RFA	28	17.6	17	32.1	1.36	0.88–2.11	
Palliative	59	37.1	8	15.1	1.00		
Biochemical characteristics							
HbA1c, % (n = 208)							0.979
≥8.5	63	40.4	18	34.6	1.00	0.73–1.34	
<8.5	93	59.6	34	65.4	1.00		
White blood cells, WBC (×10^3^/μL)							0.165
>11	29	18.2	8	15.1	1.33	0.89–2.00	
≤11	130	81.8	45	84.9	1.00		
Red blood cells, RBC (×10^12^/μL)					0.527		
High	127	79.9	47	88.7	1.00		
Low	32	20.1	6	11.3	1.13	0.60–1.30	
Haemoglobin, Hb (g/dL)						0.081	
≥12	105	66.0	43	81.1	1.00		
<12	54	34.0	10	18.9	1.34	0.97–1.86	
Platelet (×10^3^/μL)							0.694
<150	65	40.9	18	34	0.94	0.68–1.29	
≥150	94	59.1	35	66	1.00		
Mean platelet volume, MPV (fL) (n = 187)							0.359
>11	46	33.6	21	42.0	0.85	0.59–1.21	
≤11	91	66.4	29	58.0	1.00		
Albumin globulin ratio, AGR (n = 188)							0.748
<1.1	109	76.2	31	68.9	1.09	0.74–1.53	
≥1.1	34	23.8	14	31.1	1.00		
Total bilirubin (μmol/L) (n = 201)							0.020^*^
≥21	88	58.3	37	74.0	1.46	1.07–2.00	
<21	63	41.7	13	26.0	1.00		
ALP (IU/L) (n = 210)							<0.001^*^
>129	85	53.8	11	21.2	2.02	1.47–2.78	
≤129	73	46.2	41	78.8	1.00		
Alanine transaminase, ALT (IU/L) (n = 207)							0.748
≥25	123	78.8	35	68.6	1.06	0.72–1.56	
<25	33	21.2	16	31.4	1.00		
AFP (ng/mL) (n = 186)							0.012^*^
≥20	81	57.0	15	34.1	1.52	1.07–2.09	
<20	61	43.0	29	65.9	1.00		
INR (n = 173)							0.847
>1.2	32	24.1	8	20.0	1.04	0.73–1.47	
≤1.2	101	75.9	32	80.0	1.00		
